# Supplementary material for: A highly potent ruthenium(II)-sonosensitizer and sonocatalyst for in vivo sonotherapy
Source: Nat Commun. 2021 Aug 18;12:5001. doi: 10.1038/s41467-021-25303-1 (PMC8373944; doi:10.1038/s41467-021-25303-1)
Supplement: Supplementary file 1 — Supplementary Information [file 41467_2021_25303_MOESM1_ESM.pdf]

Supplementary Information

A Highly Potent Ruthenium(II)-Sonosensitizer and Sonocatalyst for *in Vivo* Sonotherapy

Chao Liang[1,2,3], Jiaen Xie[1], Shuangling Luo[1], Can Huang[4], Qianling Zhang[1], Huaiyi Huang[4], and Pingyu Zhang[1*]

[1]College of Chemistry and Environmental Engineering, Shenzhen University, Shenzhen, 518060, P. R. China.

[2]Key Laboratory of Optoelectronic Devices and Systems of Ministry of Education and Guangdong Province, College of Physics and Optoelectronic Engineering, Shenzhen University, Shenzhen 518060, P. R. China.

[3]College of Chemistry and Environmental Engineering, Hanshan Normal University, Chaozhou 521041, P. R. China.

[4]School of Pharmaceutical Sciences (Shenzhen), Sun Yat-sen University, Guangzhou, 510275, P. R. China.

Correspondence and requests for materials should be addressed to P.Z. (p.zhang6@szu.edu.cn).

Contents

Supplementary Methods

Supplementary Notes

Supplementary Figure 1. Characterization of $[Ru(bpy)_3]^{2+}$

Supplementary Figure 2. The ESR spectra of US irradiation alone.

Supplementary Figure 3. $^1O_2$ generation of $[Ru(bpy)_3]^{2+}$ measured by DPA decomposition or SOSG emission intensity increasing.

Supplementary Figure 4. Time-dependent oxidation of DPA under various experimental conditions.

Supplementary Figure 5. Time-dependent oxidation of MB to detect •OH generation by US irradiation.

Supplementary Figure 6. The sono-stability of $[Ru(bpy)_3]^{2+}$ after different US irradiation durations.

Supplementary Figure 7. The oxidation of NADH by $[Ru(bpy)_3]^{2+}$ alone or US irradiation alone.

Supplementary Figure 8. The oxidation of NADH in the presence of $[Ru(bpy)_3]^{2+}$ and NaN_3 after different US irradiation durations.

Supplementary Figure 9. Intracellular NADH sono-oxidation by $[Ru(bpy)_3]^{2+}$ under US irradiation.

Supplementary Figure 10. The cytotoxicity of $[Ru(bpy)_3]^{2+}$ with various concentrations without US irradiation or with different powers of US irradiation.

Supplementary Figure 11. The temperature curves of solution and tumor tissue under different powers of US irradiation.

Supplementary Figure 12. The cell uptake of $[Ru(bpy)_3]^{2+}$.

Supplementary Figure 13. Intracellular ROS generation of $[Ru(bpy)_3]^{2+}$ for SDT.

Supplementary Figure 14. Intracellular $O_2^{-\cdot}$ and •OH generation of $[Ru(bpy)_3]^{2+}$.

Supplementary Figure 15. *In vivo* therapeutic experiments on deep tumor model.

Supplementary Figure 16. The biosafety of $[Ru(bpy)_3]^{2+}$.

Supplementary Methods

Materials. 9,10-diphenylanthracene (DPA), 1,4-dihydronicotinamide adenine dinucleotide (NADH), 5,5-dimethyl-1-pyrroline N-oxide (DMPO), 2,2,6,6-tetramethylpiperidine (TEMP), and 5-(2,2-dimethyl-1,3-propoxycyclophosphoryl)-5-methyl-1-pyrroline N-oxide (CYPMPO) were purchased from Sigma-Aldrich. Singlet Oxygen Sensor Green reagent (SOSG), 3-(4,5-dimethylthiazol-2-yl)-2,5-diphenyltetrazolium bromide (MTT), 2′,7′-dichlorofluorescin diacetate (DCFH-DA) and cell apoptosis detection Annexin V/PI kit were obtained from Life Technologies. NAD/NADH-Glo™ kit was obtained from Promega. All other reagents were purchased from Sinopharm Chemical Reagent Co.

Instruments. ESR spectra were recorded using a Bruker Model A300 ESR spectrometer equipped with a Bruker ER 4122 SHQ resonator. NMR spectra were recorded on a Bruker AV-500MHz spectrometer. UV-visible absorption spectra were recorded on a UV-S8000 spectrophotometer. The emission spectra were recorded on an Edinburgh FS5 Fluorimeter. Confocal images were recorded on a Zeiss LSM 880 confocal microscopy. DJO-2776 sonicator was used to generate ultrasound during the treatment. The cell viability assay was recorded using a Promega microplate reader. In vivo fluorescence images were recorded using a PerkinElmer IVIS spectrum in vivo imaging system. DJO-2776 sonicator with a 5 cm^2 ultrasonic probe was used to generate ultrasound during the treatment. The ultrasound used in this article is 10% duty cycle, and the marked power is the average output power, which is calculated by multiplying the working power by 10%. Excel and Origin are used for data analysis.

Supplementary Notes

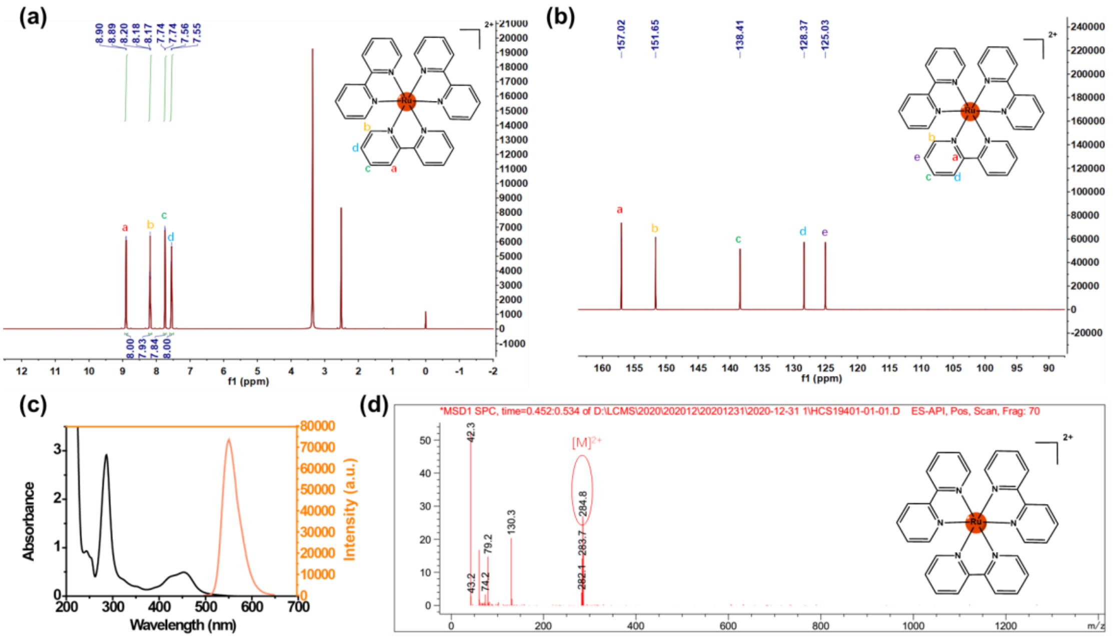

Supplementary Figure 1. Characterization of $[Ru(bpy)_3]^{2+}$. (a) ^{1}H NMR spectrum of $[Ru(bpy)_3]^{2+}$. (b) ^{13}C NMR spectrum of $[Ru(bpy)_3]^{2+}$. (c) UV-visible absorption (black) and emission (red) spectra of $[Ru(bpy)_3]^{2+}$. (d) The MS spectrum of $[Ru(bpy)_3]^{2+}$.

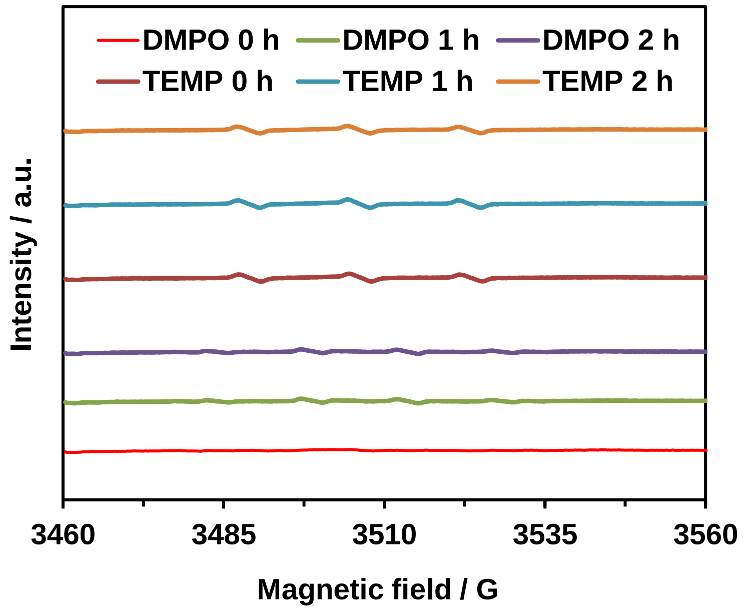

Supplementary Figure 2. The ESR spectra of US irradiation alone without $[Ru(bpy)_3]^{2+}$ in water. The TEMP and DMPO were used as $^{1}O_2$ and •OH trapping agents, respectively.

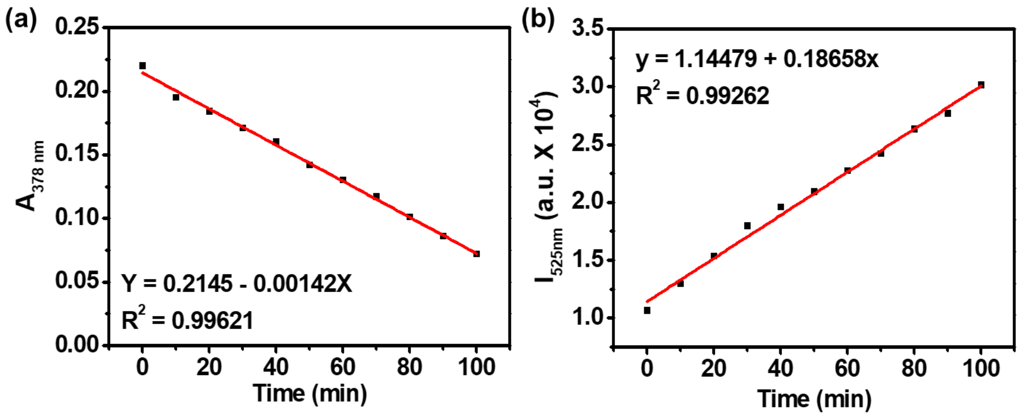

Supplementary Figure 3. (a) Rate constant for DPA decomposition at 378 nm in the presence of 10 μM $[Ru(bpy)_3]^{2+}$ under US irradiation in figure 1b. (b) The increase of SOSG emission intensity at 525 nm depending on US irradiation time in figure 1c.

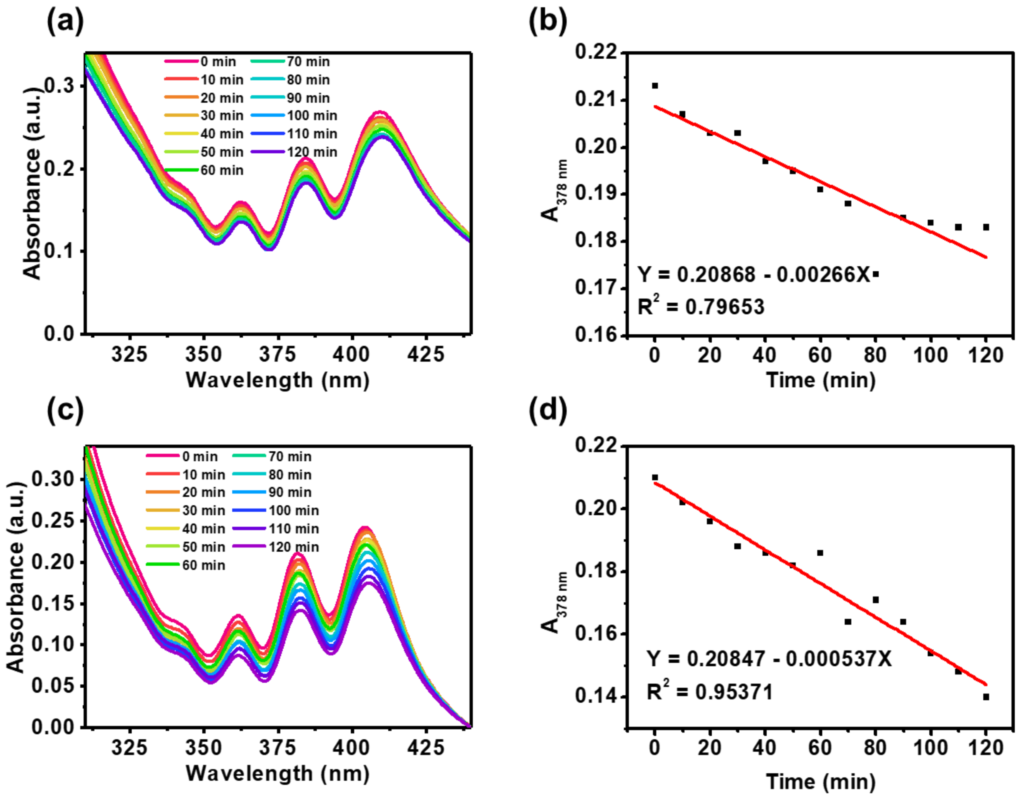

Supplementary Figure 4. Time-dependent oxidation of DPA indicating $^1O_2$ generation. (a) The absorption spectra of DPA with 10 μM $[Ru(bpy)_3]^{2+}$ at every 10 min without US irradiation. (b) Rate constant for DPA decomposition in the presence of $[Ru(bpy)_3]^{2+}$ according to the absorbance of DPA at 378 nm in (a). (c) The absorption spectra of DPA after different US irradiation durations in the absence of $[Ru(bpy)_3]^{2+}$. (d) Rate constant

for DPA decomposition under US (0.3 W cm^{-2}, 3 MHz) irradiation according to the absorbance of DPA at 378 nm in (c). These experiments were repeated three times independently with similar results.

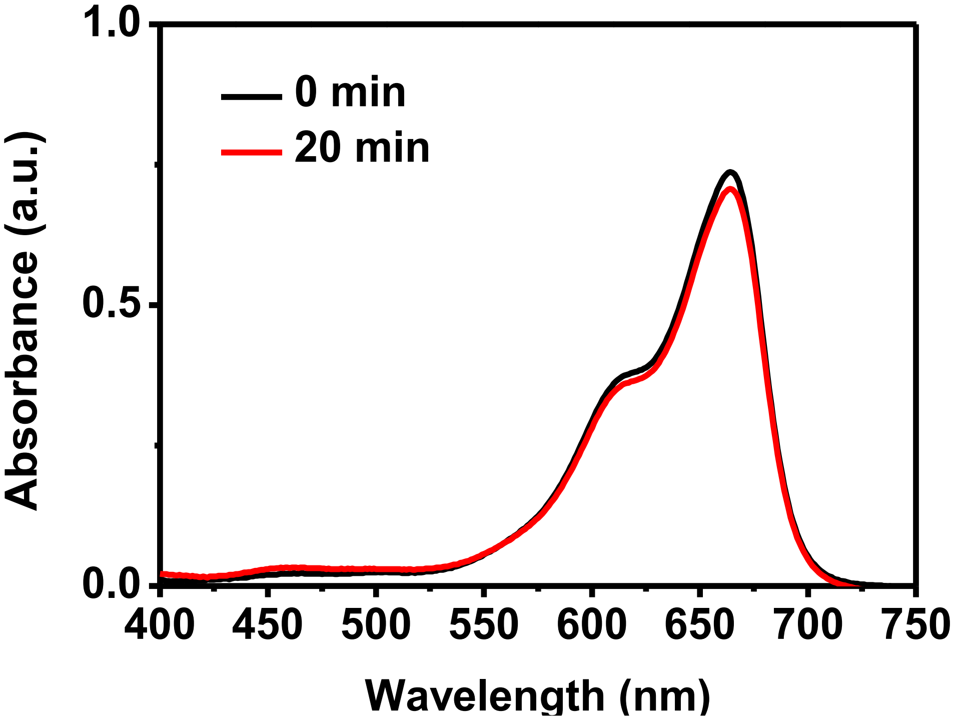

Supplementary Figure 5. Time-dependent oxidation of MB to detect •OH generation by $[Ru(bpy)_3]^{2+}$ under US irradiation. The absorption spectra of MB (5 μg mL^{-1}) in the presence of 10 μM $[Ru(bpy)_3]^{2+}$ with or without US (0.3 W cm^{-2}, 3 MHz) irradiation.

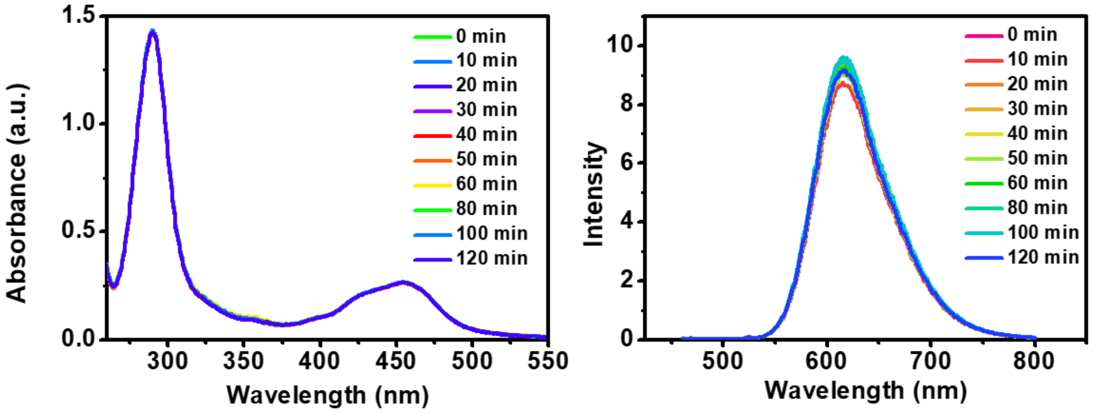

Supplementary Figure 6. Left: The absorption spectra of 10 μM $[Ru(bpy)_3]^{2+}$ after different US irradiation durations in PBS solution. Right: The emission spectra of 10 μM $[Ru(bpy)_3]^{2+}$ after different US irradiation durations in PBS solution. US irradiation: 0.3 W cm^{-2}, 3 MHz.

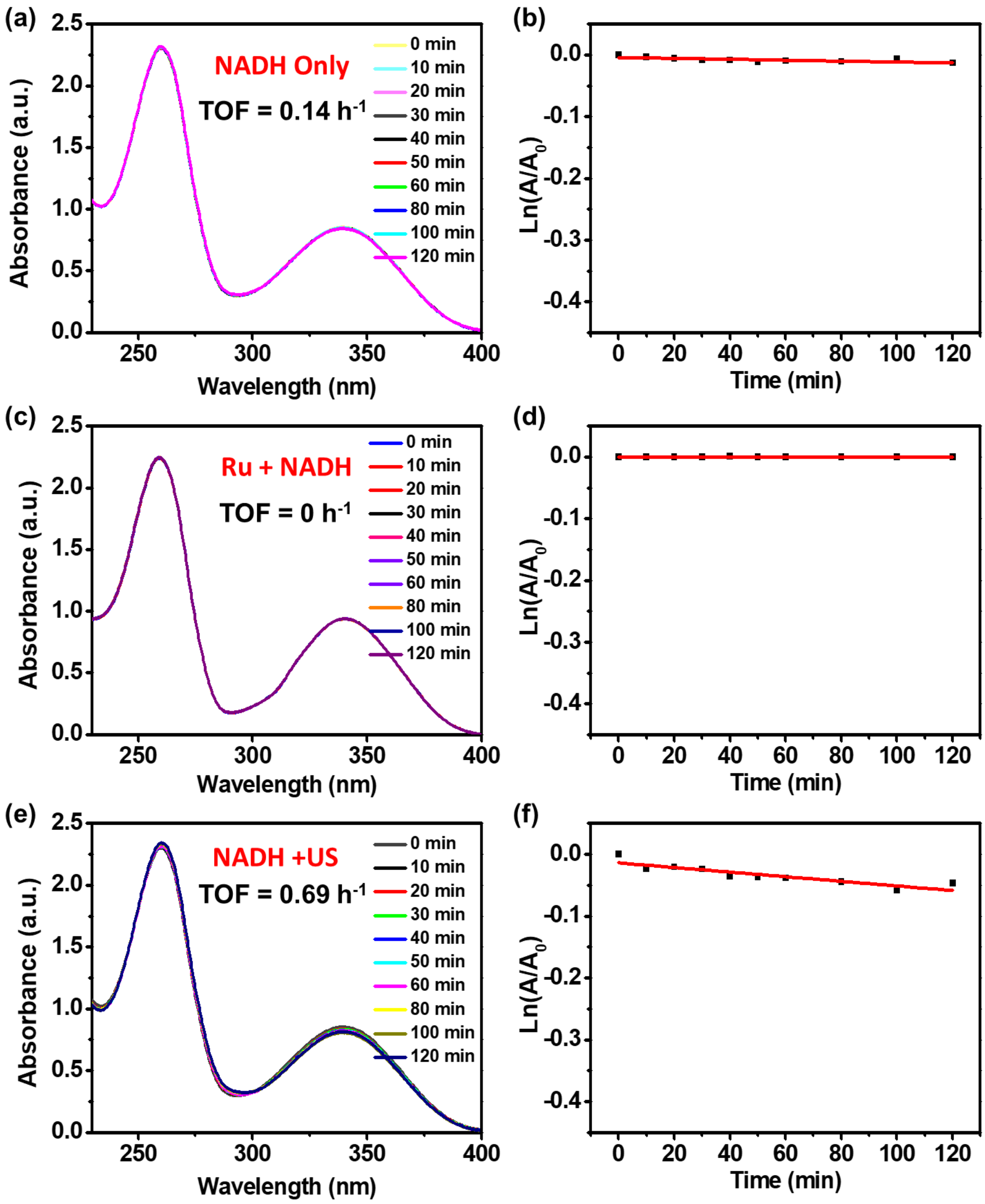

Supplementary Figure 7. The absorption spectra for oxidation of NADH by $[Ru(bpy)_3]^{2+}$ alone or US irradiation alone. (a) The absorption spectra of 150 μM NADH in PBS allowed to stand for different time. (b) Rate constant for NADH decomposition in (a) according to the absorbance of NADH at 339 nm. (c) The absorption spectra of 150 μM

NADH in PBS with 10 μM $[Ru(bpy)_3]^{2+}$ allowed to stand for different time without US irradiation. (d) Rate constant for NADH decomposition in (c) according to the absorbance of NADH at 339 nm. (e) The absorption spectra of 150 μM NADH in PBS with different US (0.3 W cm^{-2}, 3 MHz) irradiation durations. (f) Rate constant for NADH decomposition in (e) according to the absorbance of NADH at 339 nm.

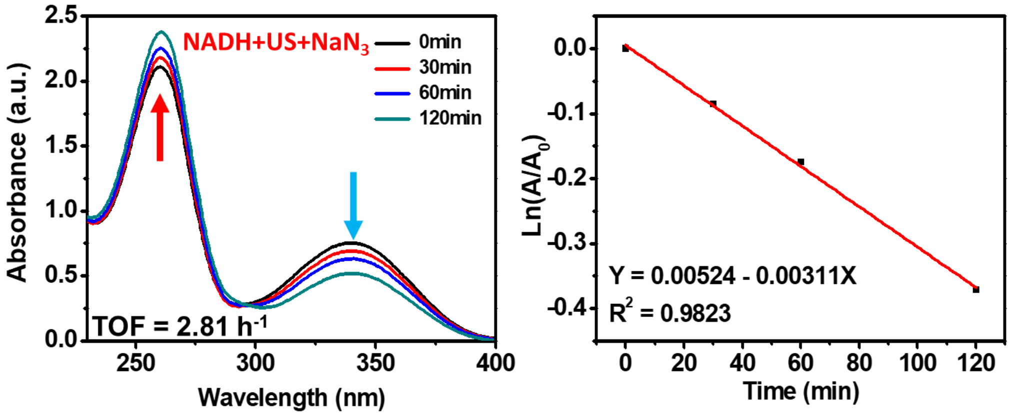

Supplementary Figure 8. The absorption spectra of 150 μM NADH in PBS in the presence of 10 μM $[Ru(bpy)_3]^{2+}$ and 1 mM NaN_3 after different US (0.3 W cm^{-2}, 3 MHz) irradiation durations. Rate constant for NADH decomposition according to the absorbance of NADH at 339 nm.

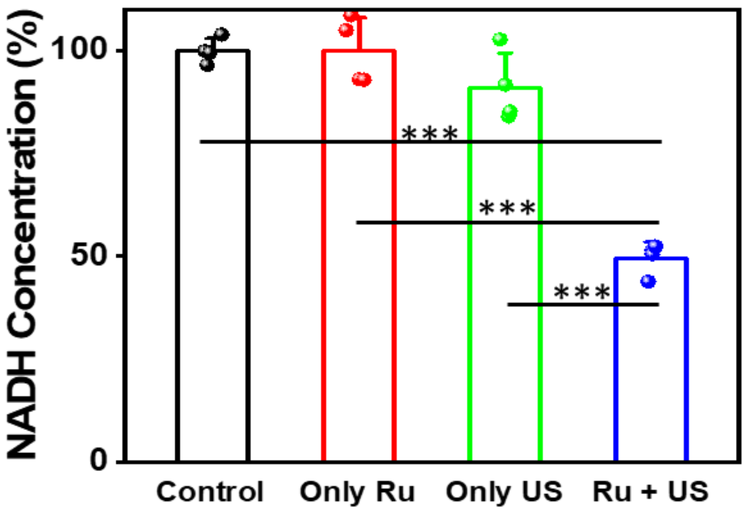

Supplementary Figure 9. Intracellular NADH oxidation by 10 μM $[Ru(bpy)_3]^{2+}$ in 4T1 cells with US (0.3 W cm^{-2}, 3 MHz, 20 min) irradiation. All the experiments were performed as duplicates of quadruplicate (n = 4 biologically independent samples). Error bars represent S.D. from the mean. Statistical significance was calculated with two-tailed Student's t test (***p < 0.001, **p < 0.01, or *p < 0.05).

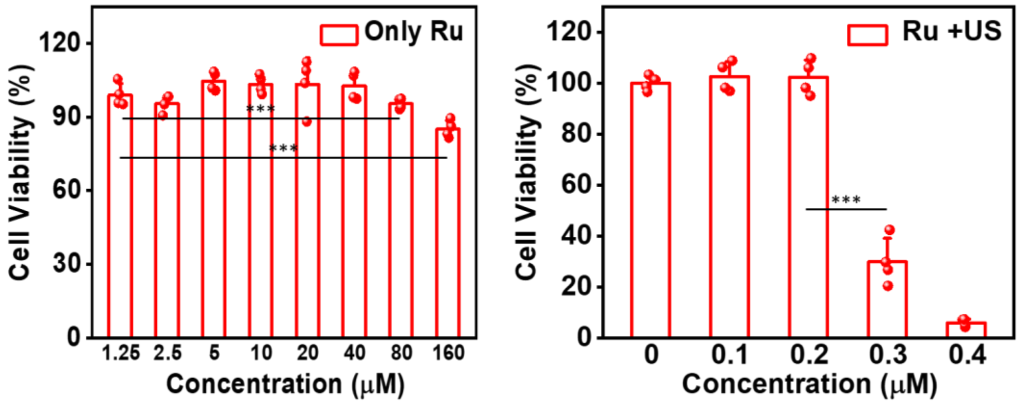

Supplementary Figure 10. (a) The dark cytotoxicity of $[Ru(bpy)_3]^{2+}$ with various concentration in 4T1 cells for 48 h. All cell viability data was performed as duplicates of quadruplicate. Error bars represent S.D. from the mean. (b) The cell viabilities of 4T1 cells treated with $[Ru(bpy)_3]^{2+}$ under varied powers of US irradiation. US irradiation: 20 min, 3 MHz. All the experiments were performed as duplicates of quadruplicate (n = 4 biologically independent samples). Error bars represent S.D. from the mean. Statistical significance was calculated with two-tailed Student's t test (***p < 0.001, **p < 0.01, or *p < 0.05).

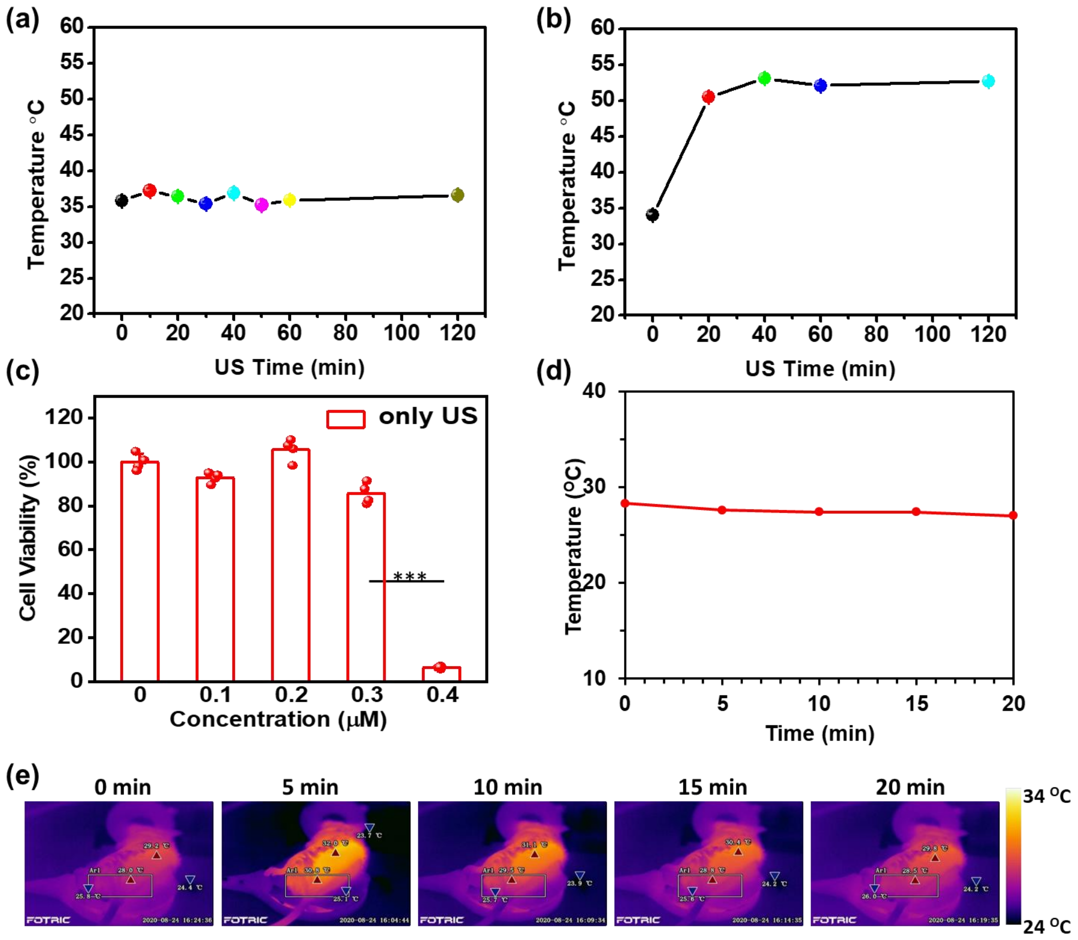

Supplementary Figure 11. The temperature change during US irradiation. (a) The temperature curve of $[Ru(bpy)_3]^{2+}$ solution during 0.3 W cm^{-2} US irradiation. (b) The temperature curve of $[Ru(bpy)_3]^{2+}$ solution during 0.4 W cm^{-2} US irradiation. (c) The cell viabilities of 4T1 cells after incubation with different US powers for 20 min. (d) The temperature curve of tumor area during SDT in (e). (e) Near infrared thermal imaging of anesthetized mice during US irradiation (0.3 W cm^{-2}, 3 MHz, 20 min). All cell viability data was performed as duplicates of quadruplicate. Error bars represent S.D. from the mean, ***$p < 0.001$, **$p < 0.01$, or *$p < 0.05$.

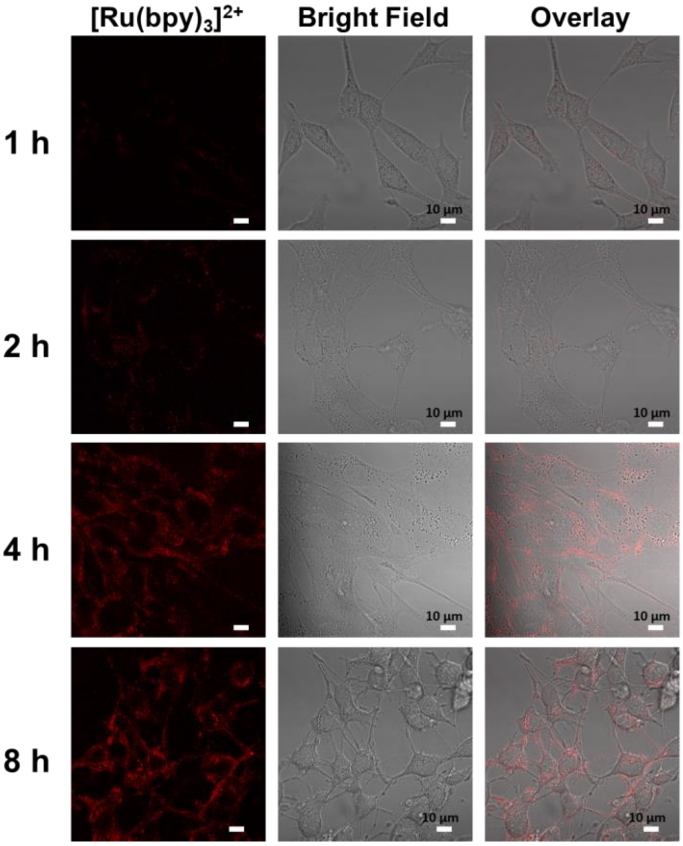

Supplementary Figure 12. The cell uptake of $[Ru(bpy)_3]^{2+}$ with different incubation time by 4T1 cells. The experiment was repeated three times independently with similar results.

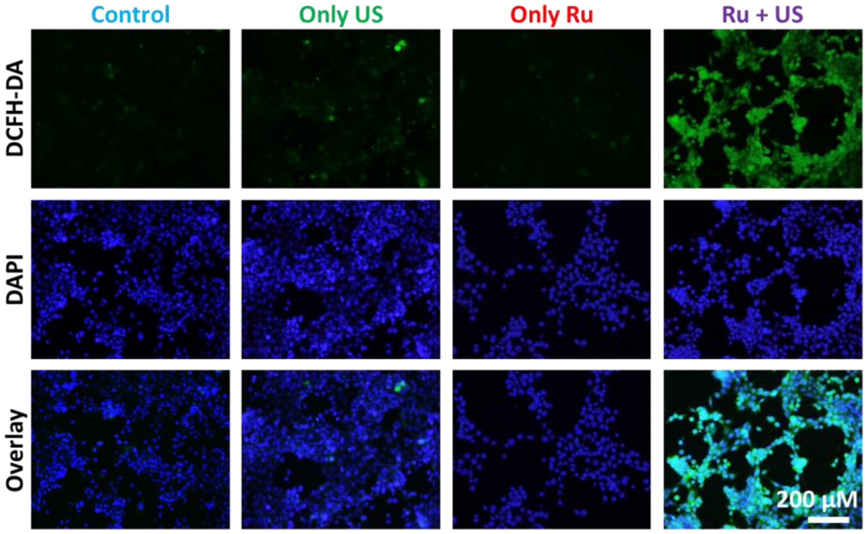

Supplementary Figure 13. Confocal images of 4T1 cells stained with DAPI (blue) and DCFH-DA (green) after various treatments. The experiment was repeated three times independently with similar results. **Ru**: 10 μM $[Ru(bpy)_3]^{2+}$; US: 0.3 W cm^{-2}, 3 MHz, 20 min.

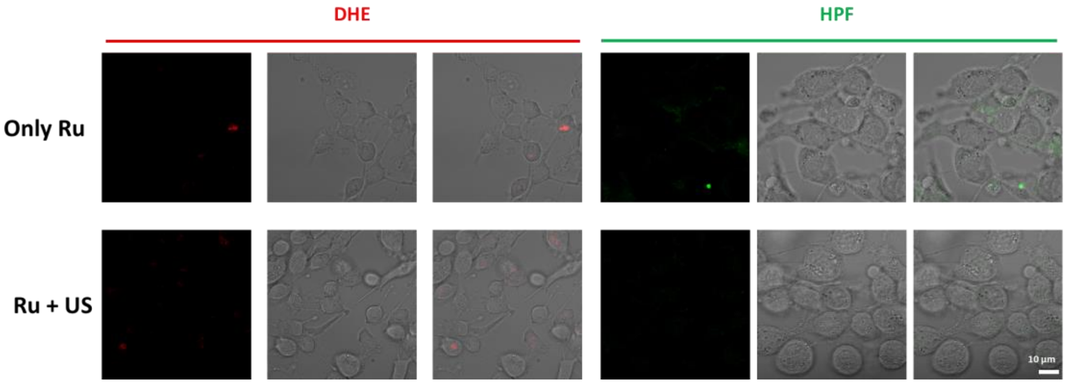

Supplementary Figure 14. Confocal images of 4T1 cells stained with DHE or HPF after various treatments. The experiment was repeated three times independently with similar results. **Ru**: 10 μM $[Ru(bpy)_3]^{2+}$; US: 0.3 W cm^{-2}, 3 MHz, 20 min.

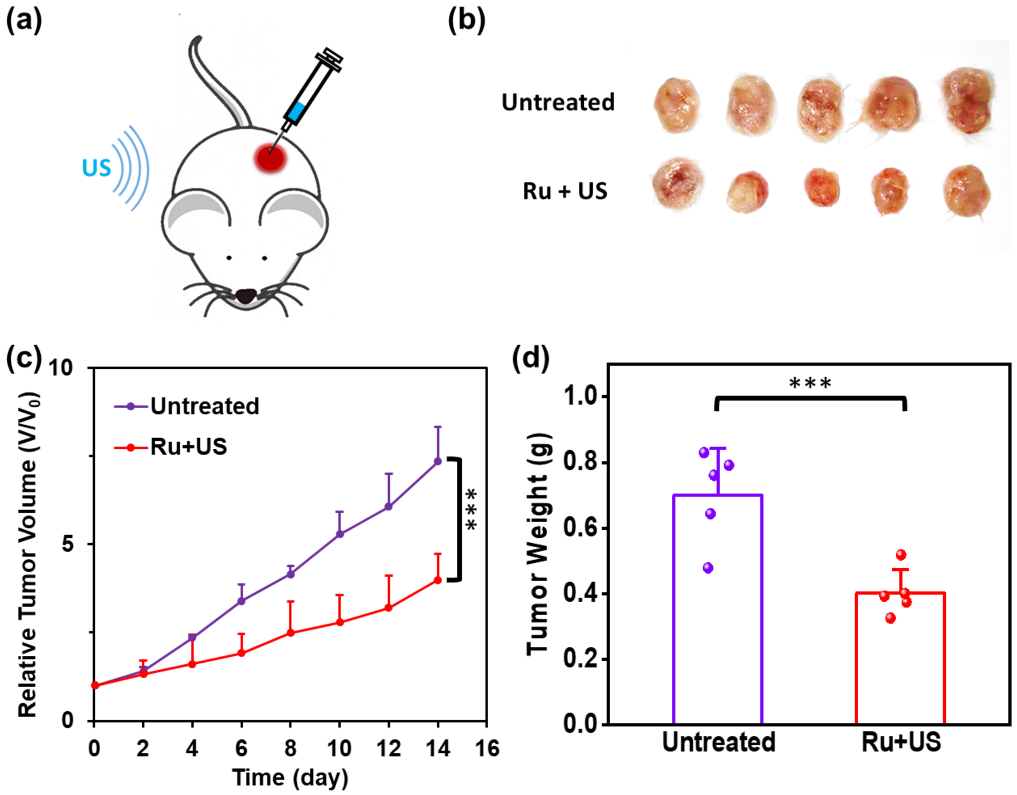

Supplementary Figure 15. *In vivo* therapeutic experiment in deep tumor model. (a) Schematic of the *in vivo* sonotherapy procedure in deep tumor model. The 4T1 tumor was transplanted on the right side of mice, and the US probe was on the left side of mice during treatment. The US waves penetrated from left side of mice to right side during SDT. (b) Photos of tumors were collected from mice at 14 day after various treatments. (c) Tumor growth curves of mice after various treatments. (d) Average tumor weights of mice at 14 day post various treatments. **Ru**: 10 μM $[Ru(bpy)_3]^{2+}$; US: 0.3 W cm^{-2}, 3 MHz, 20 min. Error bars in (c) and (d) were standard errors based on five mice in each group. Statistical significance was calculated with two-tailed Student's t test (***$p < 0.001$, **$p < 0.01$, or *$p < 0.05$).

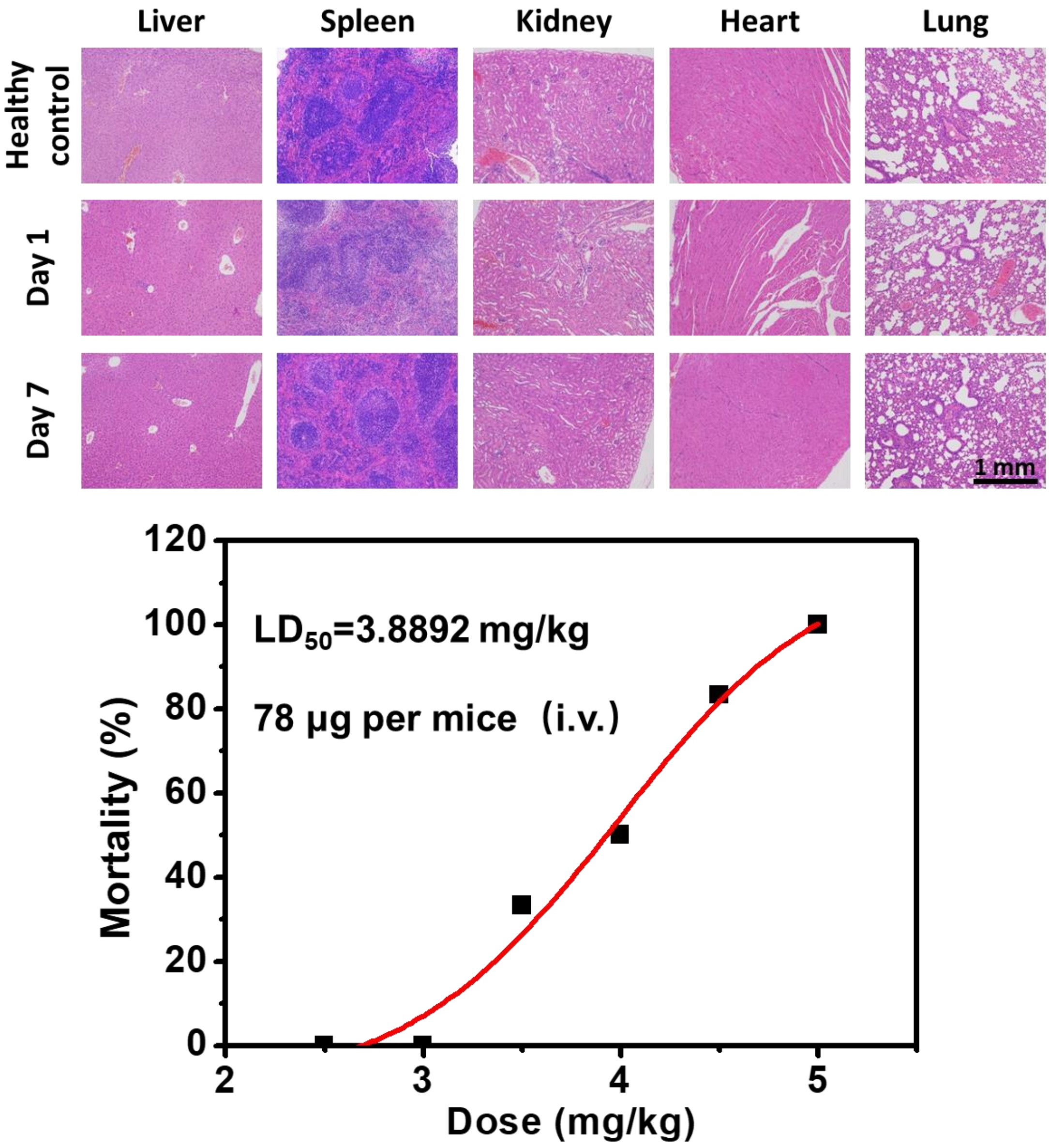

Supplementary Figure 16. The biosafety and the acute toxicity of $[Ru(bpy)_3]^{2+}$. Healthy mice were i.v. injected with 2.5 mg kg^{-1} $[Ru(bpy)_3]^{2+}$. Mice were sacrificed at 1 or 7 day post injection to collect their main organ for H&E stained slices. No obvious tissue damage can be found from these slices. In acute toxicity experiment of $[Ru(bpy)_3]^{2+}$, each i.v. injection dose was investigated in six mice. The LD_{50} of $[Ru(bpy)_3]^{2+}$ was 3.89 mg kg^{-1}.
